# Diagnostic and therapeutic challenges in non-specific orbital inflammation - a case report

**DOI:** 10.22336/rjo.2023.15

**Published:** 2023

**Authors:** Mioara-Laura Macovei, Adelina-Maria Neacșu

**Affiliations:** * Department of Ophthalmology, “Dr. Carol Davila” Central Military Emergency University Hospital, Bucharest, Romania

**Keywords:** idiopathic orbital inflammation, exposure keratopathy, hypopyon, exophthalmia

## Abstract

**Objective:** The aim of this report is to present the diagnostic and therapeutic approach in a case of non-specific orbital inflammation complicated with sight-threatening exposure keratopathy.

**Case presentation:** An 81-year-old female patient presented to our Ophthalmology Department for left, painful, unilateral exophthalmia and decreased vision in the same eye. The clinical examination revealed left proptosis, inferior dystopia, upper lid edema, erythema and moderate retraction, ocular motility restriction, chemosis and corneal epithelial defect. The keratopathy complicated in evolution with hypopyon. The patient received treatment for the exposure keratopathy and, after every specific cause of unilateral exophthalmia was ruled out using imagistic and laboratory examinations, systemic corticosteroid treatment was initiated. Although the response to treatment was prompt, it was incomplete because of the long-standing evolution.

**Discussions:** In this case, the diagnosis was difficult because a malignant breast tumor was encountered and an orbital biopsy was impossible to be performed. The presence of exposure keratopathy complicated with hypopyon imposed the exclusion of an infectious process and delayed the initiation of the corticosteroid therapy.

**Conclusions:** The diagnosis and treatment of this disease represent a challenge given the need to rule out all the local and systemic conditions that may present with exophthalmia.

## Introduction

Non-specific orbital inflammation, also known as idiopathic orbital inflammation, represents a condition with unknown etiology, characterized by inflammatory expansion of the orbital structures, potentially involving any of the soft orbital structures [**1**]. The diagnosis is one of exclusion and can be made only after all the infectious, neoplastic and specific inflammatory causes are ruled out [**2**]. The incidence of this disorder is hard to be established, some authors placing it between 8 and 11% [**3**,**4**].

The inflammation can preferentially involve certain orbital structures, such as: extraocular muscles (known as myositis), lacrimal gland (dacryoadenitis), anterior orbit or orbital apex. Another possible presentation is with diffuse inflammation of the entire orbit. Extension to intracranial structures is also described, although uncommon [**2**,**4**].

The pathophysiology of non-specific orbital inflammation remains unknown, with both infectious and immune etiologies being potentially involved. Another suggested theory refers to molecular mimicry. Recent studies have described high levels of certain interleukins (IL-2, IL-8, IL-10, IL-12), interferon-γ, and tumor necrosis factor-α cytokines, while other authors stated high expression of CD20 and CD25 [**5**].

Clinically, the unilateral presentation is typical (however, bilateral disease is described in pediatric patients, with an incidence of 8%-20% [**4**]). The most common manifestations are pain (58,3%) and periorbital swelling (79,2%) [**6**,**7**]. Periocular redness, proptosis, chemosis, limited ocular motility, ptosis, signs of optic nerve dysfunction (optic disc swelling) and choroidal folds are also described [**1**,**7**].

Imagistic investigations (oculo-orbital ultrasonography, high-resolution computed tomography or contrast-enhanced magnetic resonance imaging) usually show enlargement of involved orbital structures and other characteristic signs. B-scan ultrasonography may reveal an hypoechogenic area corresponding to the inflamed Tenon capsule (T-sign) [**2**]. CT is the method of election and it often shows enlargement of the extraocular muscle tendon insertions (unlike thyroid eye disease), or diffuse orbital densification [**8**].

The indication of biopsy is controversial and it is reserved to cases with poor response to therapy, especially to rule out neoplasia and systemic inflammatory conditions [**1**,**9**]. Histopathological examination reveals nonspecific findings, such as pleomorphic inflammatory cells (lymphocytes, plasma cells, eosinophils) and reactive fibrosis [**1**,**2**].

Systemic corticosteroids represent the main therapy and a good initial response sustains the diagnosis [**2**]. In mild cases, oral nonsteroidal anti-inflammatory drugs may be enough to control the inflammation, while resistant cases may require immunosuppressive medication (cyclosporine, methotrexate, cyclophosphamide) [**2**,**5**]. 

## Case presentation

**Fig. 1:**
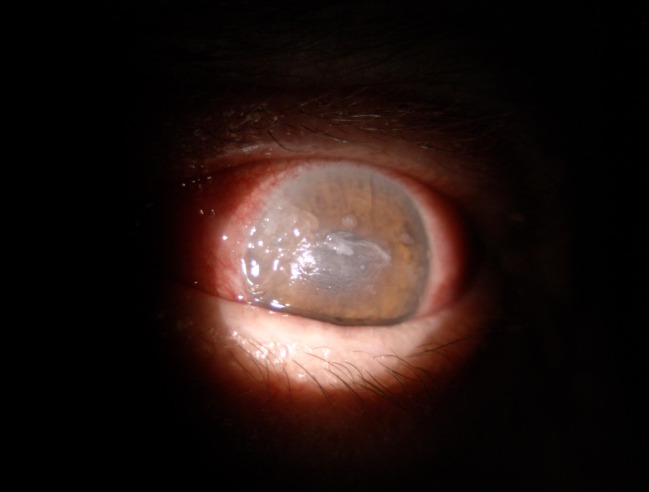
OS slit lamp image

An 81-year-old Caucasian female presented to our clinic for painful exophthalmia and decreased vision in the left eye. The patient stated that the exophthalmia had a sudden onset three months before the presentation with aggravation one month before. It was associated with pain (spontaneous and exacerbated by palpation or with eye movement), periocular redness and swelling, limitation of ocular motility and lagophthalmos. The patient reported a recent, rapid decrease in visual acuity of the affected eye, beginning one month prior to the presentation. The exophthalmia was previously diagnosed in another Ophthalmology Service as orbital cellulitis and was treated with broad spectrum systemic antibiotics, without any improvement. Past medical history revealed arterial hypertension grade II, chronic heart failure NYHA III and chronic renal failure stage 4, while past ocular history revealed senile cataract (CS 3+) and hypertensive retinopathy grade 2 in both eyes. The patient denied current ocular medication. The clinical ocular exam showed decreased visual acuity in both eyes (0.3 with best correction on the right eye and counting fingers on the left eye). The intraocular pressure was normal in OD, while OS was hypertonic at digital palpation. The external examination of the left eye revealed proptosis, inferior dystopia of 3 mm compared to OD, upper lid edema, erythema and moderate retraction, ocular motility restricted in all quadrants with severe restriction at elevation. Exophthalmometry measured 24 mm proptosis in OS (compared to 17 mm in OD, with a base of 100 mm). The slit lamp exam of the left eye unveiled mucopurulent discharge, conjunctival injection, ciliary flush, chemosis, corneal central epithelial defect that stained with fluorescein and Descemet folds. The pupil was round, central, with low pupillary response. Cortical lens opacities (3+) were encountered in both eyes (**Fig. 1**). Fundus exam showed narrowing and tortuosity of the arteries in the right eye, while the left eye could not be evaluated due to corneal defect. 

**Fig. 2:**
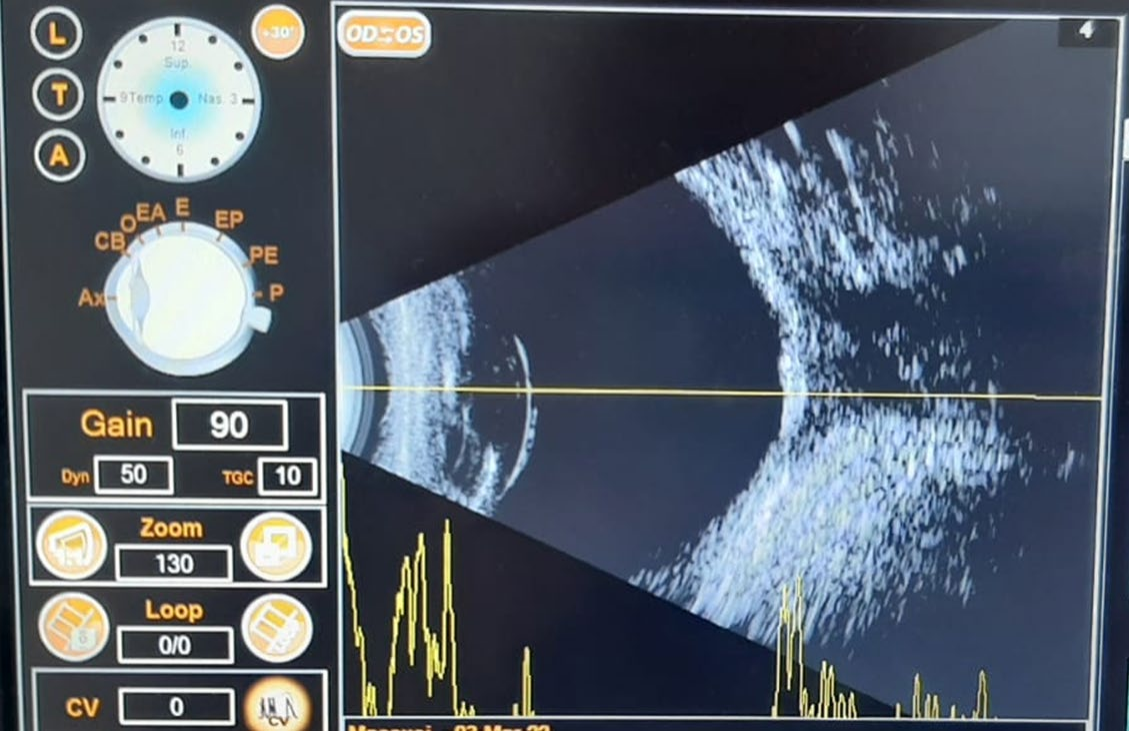
OS A, B mode echography

No echoes in the projection area of the vitreous cavity were found at the oculo-orbital ultrasonography examination (A and B mode) of the left eye, the retina seemed to be attached, while the retroorbital space appeared to be enlarged, with no space-occupying mass being detected (**Fig. 2**).

Echography also described moderate enlargement and hypo-reflectivity of extern and inferior recti muscles (including insertion). 

Cerebral CT, which was performed without contrast due to kidney failure, showed left periocular soft tissue densification, extern and inferior recti densification and possible densification of the left optic nerve. No spontaneous mixed density intracerebral lesions were described.

**Fig. 3:**
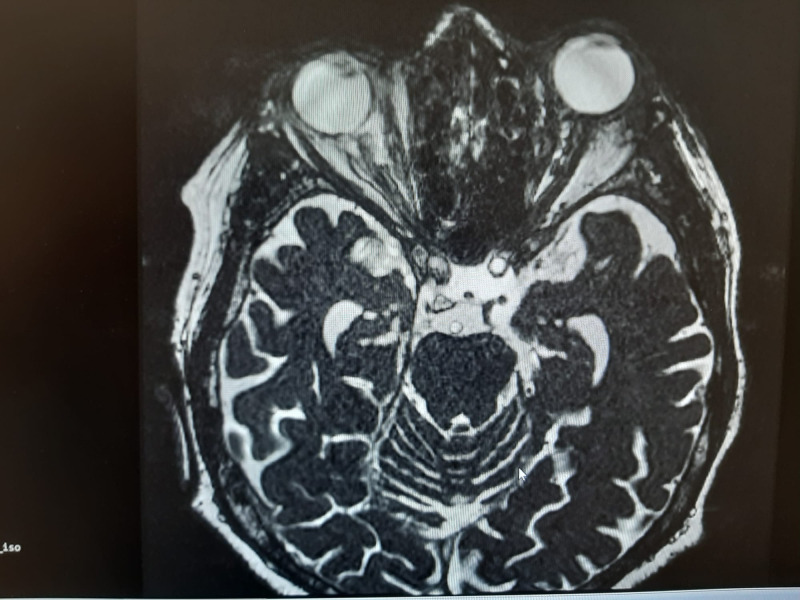
T2-weighted MRI

**Fig. 4:**
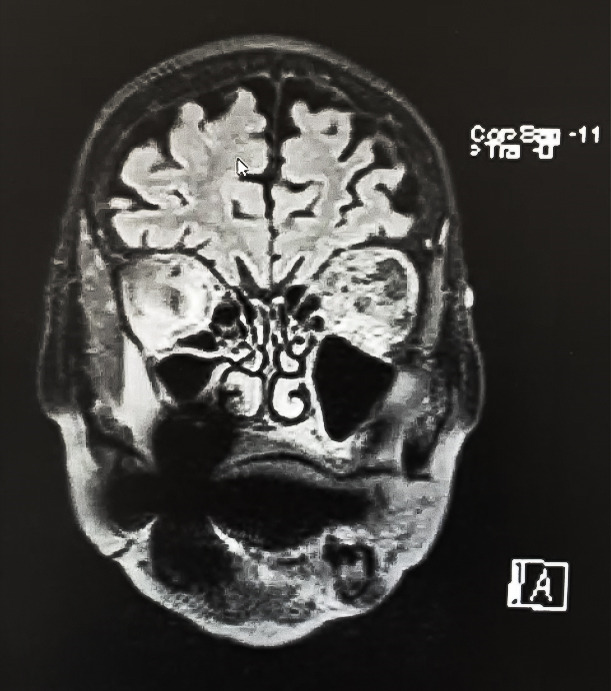
T2-weighted MRI

Cerebral MRI, also performed without contrast, revealed diffuse edema of the retrobulbar fat tissue, perineural fluid reduction - left optic nerve (mass effect), mild inflammation of the lacrimal gland and no orbital or cerebral space-occupying mass. It also confirmed the enlargement of left lateral and inferior recti muscles, including insertions (isointense in T1 and T2) (**Fig. 3**,**4**)

An extensive laboratory evaluation was initiated. The blood investigations, including complete blood count, biochemistry and coagulation tests, were modified only in the context of the preexisting renal disease.

Sedimentation rate was elevated (80 mm/h). Thyroid function was within normal limits. Further immunological tests were also performed: Angiotensin-converting enzyme (ACE), Rheumatoid factor, Antinuclear antibodies (ANA), anti-dsDNA antibodies, p-ANCA, c-ANCA, myositis autoantibodies and serum IgG4 level, each of them being in normal range. Cancer markers (CA 125, CA 15.3, CEA) proved to be elevated. Given this context, further multidisciplinary assessment was demanded, which led to the diagnosis of a right mammary tumor mass. The biopsy of the tumor revealed a lobular invasive mammary carcinoma (histological grade 2), without lympho-vascular invasion. 

Despite the discovery of this malignant process, no link between it and the left exophthalmia was proven, given the inflammatory nature of the orbital process, the imaging findings described previously and the good response to corticosteroids (that is to be further discussed). After eliminating infectious etiology, Graves orbitopathy, systemic autoimmune diseases, carotid-cavernous fistula, primary orbital tumors and orbital metastasis, a final diagnosis of left idiopathic orbital inflammatory disease was performed. The secondary ocular diagnoses were: OS-exposure keratopathy and OU-blepharoconjunctivitis, cortical senile cataract 3+, hypertension retinopathy grade 2, presbyopia.

At the initial presentation, while the etiology of the exophthalmia was unclear, the management of the exposure keratopathy and blepharoconjunctivitis was initiated with topical antibiotics (5 times daily), ocular shampoo and lubricants (5 times daily). A topical solution of a Carbonic Anhydrase Inhibitor and a Beta-blocker was administered twice daily to lower the intraocular pressure. Occlusive eye patch at night was also recommended. 

**Fig. 5:**
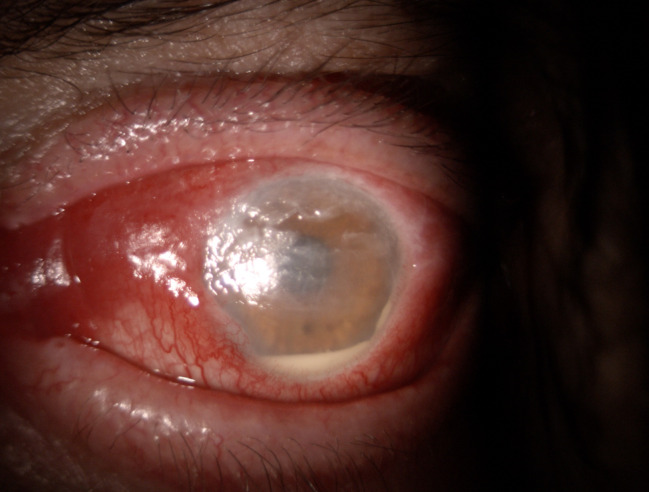
OS exposure keratopathy + hypopyon

At the 7 days follow-up, the ophthalmologic examination showed aggravation of the keratopathy in the left eye with a visual acuity of light perception, hypertonia at digital palpation, chemosis, conjunctival injection, corneal ulcer, Descemet folds, hypopyon of approximately 1 mm, low pupillary response (due to posterior iris synechiae) (**Fig. 5**). The fundus could not be explored. The patient was hospitalized for 2 weeks. Initially, the hypothesis of endophthalmitis was ruled out using ultrasonography, which revealed no vitreous echoes. After collecting corneal swabs for bacterial cultures, topical treatment with fortified antibiotics every 2 hours was initiated. In addition, the patient received topical mydriatics three times daily to reduce posterior synechiae, preservative-free lubricants and a topical combination of Beta-blocker and Carbonic Anhydrase Inhibitor to reduce intraocular pressure. An occlusive eye patch was also recommended.

Once the bacterial cultures proved to be negative, the fortified antibiotics were ceased and subconjunctival dexamethasone injections (2.5 mg) were performed once a day, for 3 days. In the meantime, the etiology of the affliction was assessed using paraclinical investigations and interdisciplinary consults and the diagnosis of idiopathic orbital inflammatory disease was established. Therefore, systemic corticotherapy was initiated, beginning with 3 days of intravenous methylprednisolone (1000 mg daily) and continuing with oral prednisone 1 mg/ kg/ day (60 mg daily) for the following 7 days, with subsequent tapering for the next 6 weeks. 

**Fig. 6:**
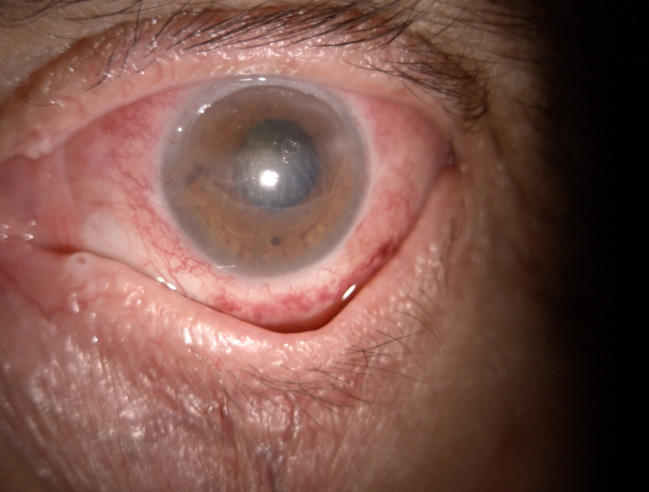
OS absent hypopyon

**Fig. 7:**
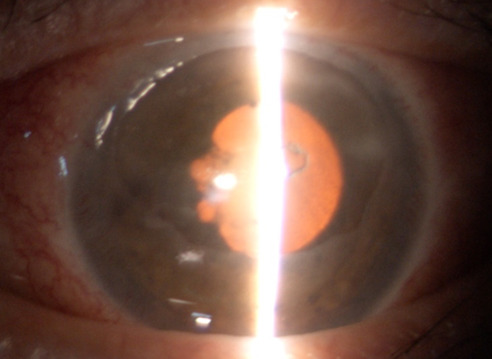
OS present red reflex

After 10 days of steroid treatment, the clinical examination of the left eye revealed a visual acuity of hand movement, normotonic eye at digital palpation, decreasing chemosis and conjunctival injection, smaller epithelial defect, resolution of the hypopyon, better pupil dilatation and better eye closure. The exophthalmia reduced by 3 mm (the Hertel exophthalmometer measured 21 mm, compared with the 24 mm measured at presentation) (**Fig. 6**,**7**).

**Fig. 8:**
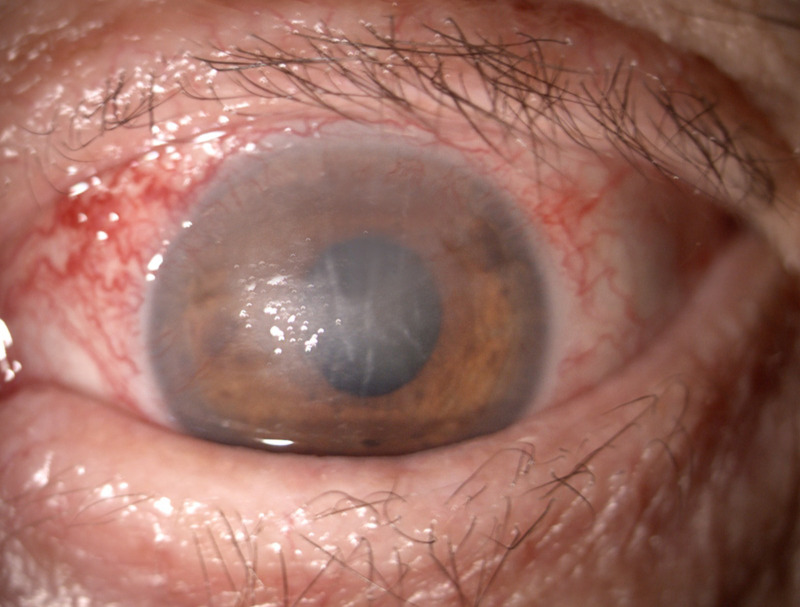
OS absent epithelial defect

**Fig. 9:**
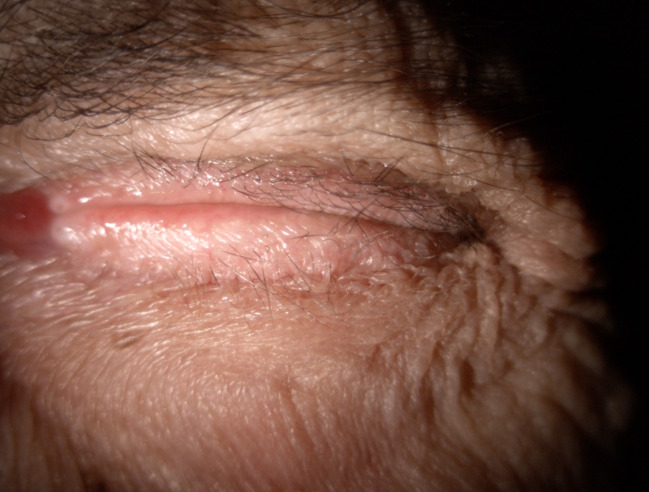
OS complete eye closure

The follow-up at the end of the steroid treatment displayed the healing of the epithelial defect. The visual acuity remained decreased (counting fingers), given the cataract and the persistent Descemet folds, while the intraocular pressure maintained normal under topical treatment. The patient was able to completely close the eye, although with effort, because of the persistent lid retraction. Exophthalmometry showed additional proptosis reduction with only 1 mm (**Fig. 8**,**9**).

## Discussion

The patient was lost to follow-up due to aggravation of the systemic conditions.

Given the fact that the diagnosis of this condition is one of exclusion, the differential diagnosis with all the causes of unilateral exophthalmia led to the delaying of the treatment. 

The presence of a potentially metastatic malignant breast tumor made the diagnosis more difficult since the patient refused the orbital biopsy. In this situation, this possibility was eliminated based on clinical presentation (rapid onset, no palpable mass or subcutaneous nodules, local inflammation and pain), imaging (no unencapsulated orbital mass, no bone erosion and homogenous MRI aspect of the retroorbital tissue), and therapeutic trial (rapid response to corticosteroid therapy). Furthermore, the mammary tumor biopsy revealed no lympho-vascular invasion.

Moreover, the systemic corticosteroid treatment in this case was delayed by the presence of the severe exposure keratopathy complicated with hypopyon. In this case, the exclusion of an infection was compulsory.

As a result, the response to corticosteroid administration was not complete, probably due to fibrosis, developed during the long-standing history of the disease (3 months). An incomplete response to therapy is not uncommon in IOI, a study conducted on 27 patients showing a cure rate of only 37%, despite an initial good response of 78% [**10**]. In this situation, further therapy with low dose local radiotherapy may lead to the resolution of the exophthalmia [**11**].

## Conclusions

In conclusion, idiopathic orbital inflammation remains a diagnostic and therapeutic challenge, given the wide range of pathologies that must be excluded, the sight-threatening complications and the poor response to current therapeutic means. The aim of this report was to highlight the difficulties encountered in managing a long-standing case, in which the presence of a malignant breast tumor made the differential diagnosis harder.

**Conflict of Interest Statement**

The authors state no conflict of interest.

**Informed Consent and Human and Animal Rights statement**

An informed consent was obtained from the patient included in the case report.

**Authorization for the use of human subjects**

Ethical approval: The research related to human use complies with all the relevant national regulations, institutional policies, it is in accordance with the tenets of the Helsinki Declaration and has been approved by the review board of Ophthalmology Department, “Dr. Carol Davila” Central Military Emergency University Hospital, Bucharest, Romania. 

**Acknowledgements**

None.

**Sources of Funding**

None.

**Disclosures**

None.

**Contribution**

Both authors contributed equally to this article.
